# Inhibiting miRNA in *Caenorhabditis elegans *using a potent and selective antisense reagent

**DOI:** 10.1186/1758-907X-1-9

**Published:** 2010-04-01

**Authors:** Genhua Zheng, Victor Ambros, Wen-hong Li

**Affiliations:** 1 Departments of Cell Biology and of Biochemistry, University of Texas Southwestern Medical Center, 5323 Harry Hines Blvd, Dallas, TX 75390-9039, USA; 2 Program in Molecular Medicine, University of Massachusetts Medical School, Worcester, MA 01605, USA

## Abstract

**Background:**

Antisense reagents can serve as efficient and versatile tools for studying gene function by inhibiting nucleic acids *in vivo*. Antisense reagents have particular utility for the experimental manipulation of the activity of microRNAs (miRNAs), which are involved in the regulation of diverse developmental and physiological pathways in animals. Even in traditional genetic systems, such as the nematode *Caenorhabditis elegans*, antisense reagents can provide experimental strategies complementary to mutational approaches. Presently no antisense reagents are available for inhibiting miRNAs in the nematode *C. elegans*.

**Results:**

We have developed a new class of fluorescently labelled antisense reagents to inhibit miRNAs in developing worms. These reagents were synthesized by conjugating dextran with 2'-O-methyl oligoribonucleotide. The dextran-conjugated antisense reagents can be conveniently introduced into the germline of adult hermaphrodites and are transmitted to their progeny, where they efficiently and specifically inhibit a targeted miRNA in different tissues, including the hypodermis, the vulva and the nervous system. We show that these reagents can be used combinatorially to inhibit more than one miRNA in the same animal.

**Conclusion:**

This class of antisense reagents represents a new addition to the toolkit for studying miRNA in *C. elegans*. Combined with numerous mutants or reporter stains available, these reagents should provide a convenient approach to examine genetic interactions that involve miRNA, and may facilitate studying functions of miRNAs, especially ones whose deletion strains are difficult to generate.

See related research article: http://jbiol.com/content/9/3/20

## Background

MicroRNAs (miRNAs) are single strand RNA molecules ~ 21-23 nucleotides long that play important roles in many biological processes through regulating gene expression [1]. In animal cells, miRNAs act primarily by inhibiting mRNA translation and/or stability through a process involving partial complementary base-pairing with sequences at the 3'-untranslated region (3' UTR). Numerous miRNAs have been identified. To study their functions, antisense reagents against miRNAs have been developed as a reverse genetics tool. Synthetic oligonucleotide analogues, including 2'-O-methyl oligoribonucleotides [2], locked nucleic acids [3], 2'-O-methoxyethyl oligoribonucleotides [4], and morpholinos [5], have been tested. These antisense nucleotide analogues have been used to knock down miRNAs in cultured cells [2-4] and in live animals including zebrafish [5], *D. melanogaster *[6] and mice [7].

*Caenorhabditis elegans *has long been used as a model organism for studying the regulation and function of small non-coding RNA molecules, and yet no antisense reagents are available to reliably inhibit miRNAs in worms. Such a technique would be very useful for studying functions of miRNAs whose deletion strains are difficult to generate; for example, mutations causing lethality or sterility [8]. In addition, to dissect functions of individual miRNAs that are clustered together, or to block intronic miRNAs [8,9] without perturbing the function of the corresponding protein-coding genes, antisense reagents would offer a convenient approach to circumvent the limitation of using deletion strains.

## Results and discussion

**Figure 1:**
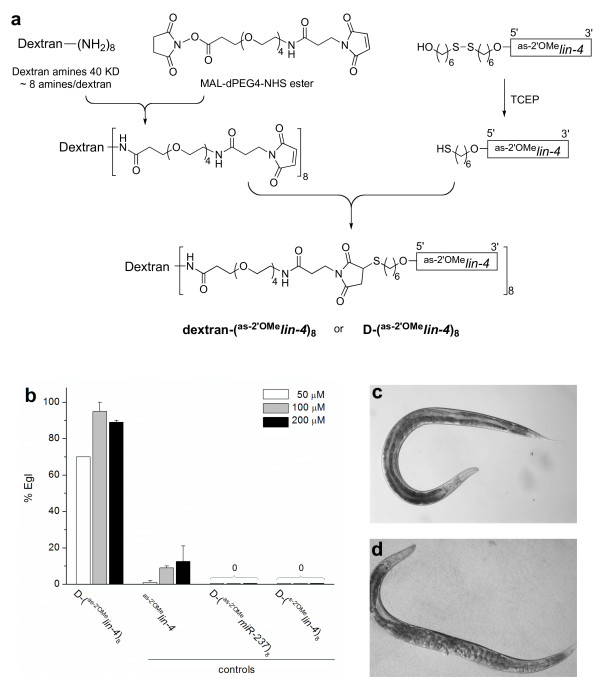
**Inhibit miRNA in *Caenorhabditis elegans* using a dextran-conjugate of antisense 2'-*O*-methyl oligoribonucleotide**. (a) Chemical synthesis of dextran-(^as-2'OMe^*lin-4*)_8_, or D-(^as-2'OMe^*lin-4*)_8_, a dextran conjugate of an antisense reagent against *lin-4*. (b) Dose response of D-(^as-2'OMe^*lin-4*)_8 _in causing egg laying defectives (Egl) via *lin-4 *inhibition. Controls include ^as-2'OMe^*lin-4*, a *lin-4 *antisense 2'-*O*-methyl oligoribonucleotide without dextran; D-(^s-2'OMe^*lin-4*)_8 _and D-(^as-2'OMe^*miR-237*)_8_, dextran conjugates containing either *lin-4 *sense or *miR-237 *antisense 2'-*O*-methyl oligoribonucleotide. Error bars are standard errors of two independent injections. Each time ~ eight worms were injected and 50 or more labelled embryos from injected worms were scored for Egl when they reached adults. (c, d) Example images of a normal worm (c) and an Egl worm (d) labelled with dextran-(^as-2'OMe^*lin-4*)_8_.

Previous studies by Hutvagner *et al. *showed that antisense 2'-O-methyl oligoribonucleotide injected into *C. elegans *larvae could inhibit functions of a miRNA [2]. However, injection of worm larvae is technically very demanding, and so the larval injection of anti-microRNA oligonucleotides has not been employed for *C. elegans *except the original report [2]. A much more straightforward approach would be to inject the antisense compound into the syncitial gonad of hermaphrodites using the standard injection methods employed for the DNA transformation of *C. elegans *[10] so that the reagent would inhibit a targeted microRNA during the embryonic and larval development of the injected hermaphrodite's progeny. A previous attempt at this approach using 2'-O-methyl oligoribonucleotides failed to produce the expected phenotype [2]. We suspect that the cellular uptake, retention or distribution properties of unconjugated 2'-O-methyl oligoribonucleotides might not be optimal for an efficient inhibition of miRNA in worms. To develop a robust antisense technique to inhibit miRNAs in *C. elegans*, we explored modifying 2'-O-methyl oligoribonucleotides through conjugation with dextran, a polysaccharide that is nontoxic, inert and soluble in aqueous solutions. Dextrans are well retained in cells over a long period of time and dextran-dye conjugates have been widely used for cell labelling and cell lineage tracing [11]. To conjugate dextran with 2'-O-methyl oligoribonucleotides, we first reacted dextran amines (molecular weight 40 KDa, ~ 8 amines/dextran) with a water-soluble bifunctional linker, MAL-dPEG4-NHS ester in order to produce dextran- [(PEG)_4_-MAL]_8 _(Figure 1a). This thiol-reactive intermediate was then conjugated with eight equivalents of antisense 2'-O-methyl oligoribonucleotides containing a 5'-thiol group. The resulting product, dextran-(^as-2'OMe^*lin-4*)_8_, or D-(^as-2'OMe^*lin-4*)_8_, contains, on average, eight copies of 2'-O-methyl oligoribonucleotides complementary to *lin-4*, the founding miRNA first identified in *C. elegans *[12,13].

To apply dextran-(^as-2'OMe^*lin-4*)_8 _to inhibit *lin-4 in vivo*, we injected the compound into gonads of adult hermaphrodites. Dextran-rhodamine (40 KDa) was coinjected as a fluorescent marker. About 16 h after injection, we collected rhodamine-labeled embryos (*n *= 50) under a fluorescence dissection scope. When these embryos reached adulthood, we scored for the egg laying defective (Egl) phenotype. In *C. elegans*, *lin-4 *is required during larval development to control the timing and pattern of cell division in the hypodermis of larva stage 1 (L1) and stage 2 (L2). *lin-4 *loss of function mutants (*lin-4*(*lf*)) display inappropriate reiterations of early fates at late developmental stages and show a retarded heterochronic phenotype in adults in the form of the absence of adult structures (such as vulva) and the failure of egg-laying [12,13].

When using an injection with a concentration of 50 μM (all concentrations refer to the total concentration of 2'-O-methyl oligoribonucleotides in the sample as determined from the ultraviolet (UV) absorption at 260 nm) dextran-(^as-2'OMe^*lin-4*)_8 _was effective in inhibiting *lin-4 *and caused Egl in about 70% of worms (Figure 1b-d). Raising the injection concentration to 100 μM or above increased Egl to over 90% in the labelled worms. In contrast, antisense 2'-O-methyl oligoribonucleotides that is not conjugated to dextran only had a small effect, even at 200 μM (Figure 1b). In order to examine the specificity of dextran-(^as-2'OMe^*lin-4*)_8 _in inhibiting *lin-4*, we prepared two control dextran conjugates, dextran-(^as-2'OMe^*miR-237*)_8 _and dextran-(^s-2'OMe^*lin-4*)_8_. Dextran-(^as-2'OMe^*miR-237*)_8 _contains 2'-O-methyl oligoribonucleotides complementary to *miR-237*, a miRNA of the *lin-4 *family with similar, but not identical, sequence as *lin-4*. Dextran-(^s-2'OMe^*lin-4*)_8 _contains *lin-4 *sequence (sense). We did not observe Egl phenotypes, or other abnormalities, in worms labelled with either of these two control oligonucleotides (Figure 1b) which confirmed that dextran-(^as-2'OMe^*lin-4*)_8 _inhibits *lin-4 *in a sequence specific manner, also suggesting that worms tolerate dextran conjugates of 2'-O-methyl oligoribonucleotides fairly well.

**Figure 2:**
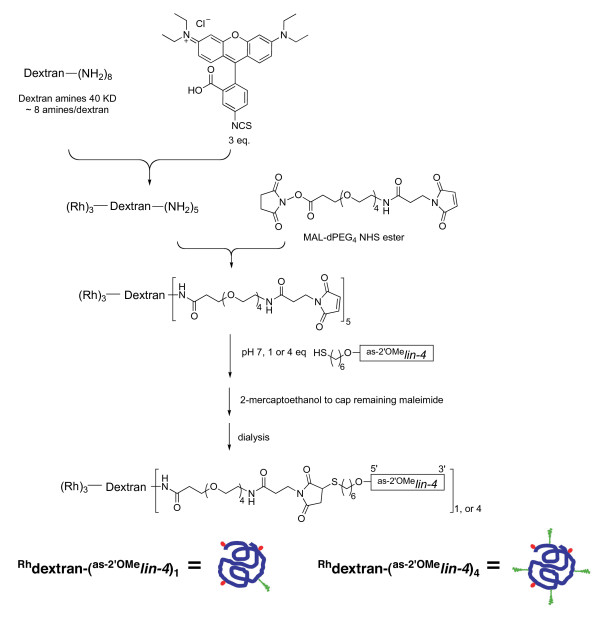
**Synthesis of rhodamine labelled dextran conjugates of 2'-*O*-methyl oligoribonucleotide**. During conjugation, different equivalents of oligoribonucleotides were used to couple with dextran to yield ^Rh^dextran-(^as-2'OMe^*lin-4*)_1 _or ^Rh^dextran-(^as-2'OMe^*lin-4*)_4_. Schematic structures of these products are shown at the bottom, with the heavy blue line, wavy green line and the red dot representing dextran, 2'-*O*-methyl oligoribonucleotide and rhodamine, respectively. Rh = rhodamine; ^Rh^Dextran = rhodamine labeled dextran.

**Figure 3:**
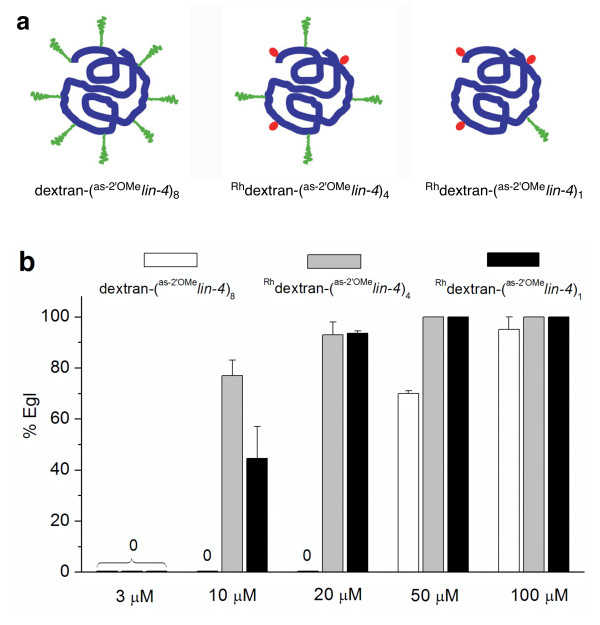
**Coupling stoichiometry of 2'-*O*-methyl oligoribonucleotide affects the potency of dextran-conjugated antisense reagents**. (a) Schematic structures of dextran-(^as-2'OMe^*lin-4*)_8_, ^Rh^dextran-(^as-2'OMe^*lin-4*)_4 _and ^Rh^dextran-(^as-2'OMe^*lin-4*)_1_. The heavy blue line, wavy green line and the red dot symbolize dextran, 2'-*O*-methyl oligoribonucleotide and rhodamine, respectively. (b) Dose response of dextran-(^as-2'OMe^*lin-4*)_8_, ^Rh^dextran-(^as-2'OMe^*lin-4*)_4_, and ^Rh^dextran-(^as-2'OMe^*lin-4*)_1 _in causing egg laying defectives. Error bars are standard errors of two independent experiments. Each time we scored at least 50 worms labelled with an antisense reagent.

In dextran-(^as-2'OMe^*lin-4*)_8_, each dextran molecule is conjugated to eight copies of antisense 2'-O-methyl oligoribonucleotides. Having a high density of oligonucleotides on the surface of dextran molecule might increase the steric hindrance and compromise the hybridization efficiency of the antisense oligonucleotide to its target miRNA. In order to test whether we could improve the potency of these dextran conjugated antisense reagents by varying the coupling stoichiometry, we decreased the amount of 2'-O-methyl oligoribonucleotides used for conjugation. In addition, we also linked a fluorescent label (rhodamine B isothiocyanate) with dextran so that we could visualize the distribution of these antisense reagents directly. We prepared two rhodamine-dextran (^Rh^dextran) conjugates of 2'-O-methyl oligoribonucleotides, ^Rh^dextran-(^as-2'OMe^*lin-4*)_4 _and ^Rh^dextran-(^as-2'OMe^*lin-4*)_1_, by varying the equivalents of 2'-O-methyl oligoribonucleotides added to the conjugation reaction (Figure 2). Each ^Rh^dextran-(^as-2'OMe^*lin-4*)_4 _or ^Rh^dextran-(^as-2'OMe^*lin-4*)_1 _on average contains four or one copy of *lin-4 *antisense 2'-O-methyl oligoribonucleotide, respectively (Figures 2 and 3a). These two dextran conjugates were comparably efficient in inhibiting *lin-4*, yet both were much more potent than dextran-(^as-2'OMe^*lin-4*)_8_. At 20 μM or above, both ^Rh^dextran-(^as-2'OMe^*lin-4*)_4_and ^Rh^dextran-(^as-2'OMe^*lin-4*)_1 _caused Egl in nearly 100% of labelled worms (Figure 3b). In contrast, dextran-(^as-2'OMe^*lin-4*)_8 _was completely ineffective at 20 μM. In addition, fluorescence imaging of ^Rh^dextran-(^as-2'OMe^*lin-4*)_1 _confirmed that the conjugate was localized fairly evenly in the cytosol after being taken up by cells.

In order to confirm that these antisense reagents act specifically by inhibiting *lin-4*, we examined several molecular and cellular markers to characterize the development of animals labelled with ^Rh^dextran-(^as-2'OMe^*lin-4*)_1_: (1) the formation of vulval structure; (2) adult specific alae formation and *col-19 *expression; and (3) the stage-specific seam cell division programmes.

**Figure 4:**
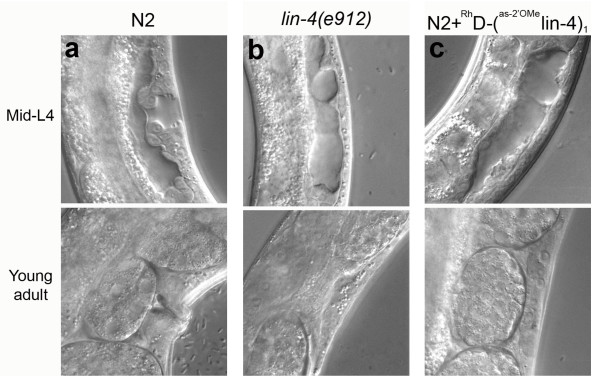
***lin-4 *inhibition with ^Rh^dextran-(^as-2'OMe^*lin-4*)_1_disrupted vulva formation similarly as in a *lin-4(0) *mutant**. The vulval structures in mid-L4 (top) and in adult (bottom) are well defined in control animals (a). Vulva failed to form in *lin-4(e912) *(b) and in *lin-4 *knockdown (c). The penetrance of vulval defect in (c) was 100% (*n *= 45) with 50 μM of ^Rh^dextran-(^as-2'OMe^*lin-4*)_1_.

The egg-laying defect of *lin-4(lf) *was due to inappropriate vulva development. In control animals, vulva morphogenesis is evident by early L4. By mid-L4 stage, the developing vulva displays a characteristic structure reminiscent of a Christmas tree (Figure 4a). In contrast, the vulva structure was missing in a *lin-4*(0) mutant, *lin-4(e912) *(Figure 4b). Wild type animals labelled with ^Rh^dextran-(^as-2'OMe^*lin-4*)_1 _showed the similar vulvaless anatomy as *lin-4(e912) *(Figure 4c), consistent with *lin-4 *inhibition by ^Rh^dextran-(^as-2'OMe^*lin-4*)_1_.

**Figure 5:**
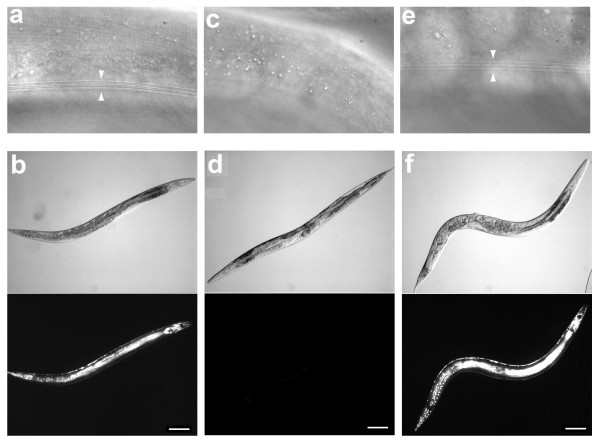
**Inhibition of *lin-4 *with ^Rh^dextran-(^as-2'OMe^*lin-4*)_1 _led to retarded development**. (a, b) Control young adult animals (~ 55 h post-hatching) showed normal alae [(a) highlighted by arrow heads] and col-19::GFP expression [(b) top - bright field; bottom - green fluorescence protein (GFP)]. (c-f) Young adult animals (~ 55 h post-hatching) labelled with ^Rh^dextran-(^as-2'OMe^*lin-4*)_1 _(50 μM injection concentration) displayed no alae (c) or col-19::GFP (d). 10 hours later (~ 65 h post-hatching), both alae (e) and col-19::GFP (f) appeared in these animals. Scale bar = 0.1 mm.

Besides affecting the development of vulval cells, *lin-4 *also controls stage-specific lateral hypodermal cell fates. At the L4 molt, lateral seam cells exit cell cycle and form alae. At about the same time, hypodermal cells start to express an adult specific green fluorescence protein (GFP) reporter, col-19::GFP (Figure 5a, b) [14,15]. Animals labelled with ^Rh^dextran-(^as-2'OMe^*lin-4*)_1 _(50 μM injection concentration) failed to form alae at the L4 molt (100%, *n *= 20) and showed no col-19::GFP expression (100%, *n *= 56; Figure 5c, d). Interestingly, 10 h later, both col-19::GFP (100%, *n *= 56) and alae (100%, *n *= 19) were observed in these animals (Figure 5e, f). Increasing the dose of ^Rh^dextran-(^as-2'OMe^*lin-4*)_1 _(150 μM injection concentration) prolongs the duration of *lin-4 *inhibition, such that at 10 h post-L4 molt, only 1.8% of labelled worms showed col19::GFP (*n *= 55); about 60% of these animals displayed col19::GFP 15 h later (or 25 h post-L4 molt). These results suggest that *lin-4 *knockdown with ^Rh^dextran-(^as-2'OMe^*lin-4*)_1 _caused these animals to go through extra larval stages (L5 and L6) before they entered adult. This is consistent with a reduction in (but not complete elimination of) *lin-4 *function.

**Figure 6:**
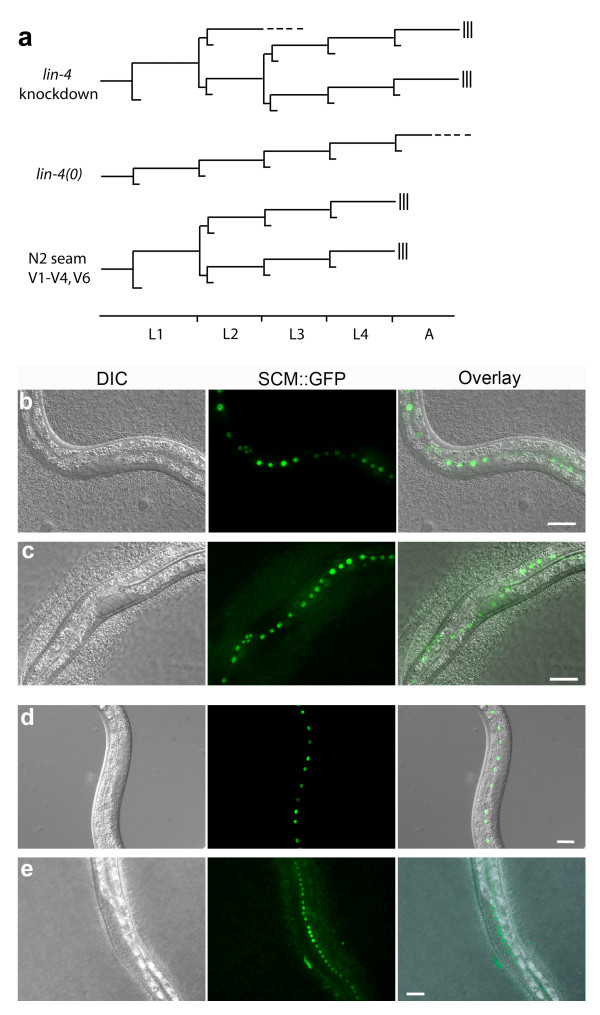
***lin-4 *knockdown caused repetition of L2 seam cell division pattern in L3**. (a) Seam cell lineage for V1 - V4 and V6 of wild type, *lin-4(0) *and *lin-4 *knockdown by ^Rh^dextran-(^as-2'OMe^*lin-4*)_1 _at 50 μM injection concentration. (b, c) In early L2, each N2 seam cell gave rise to a quartet of daughter cells after two successive divisions in both N2 (b) and in *lin-4 *knockdown (c). (d, e) In early L3, each Vn cell of *lin-4 *knockdown repeated L2 programme, giving rise to twice as many daughter cells (e) as those of control animals (d). Each image is representative of at least six animals (see text). Scale bar = 20 μm.

During larval development, the lateral hypodermal seam cells follow a characteristic division pattern. After each division, one of the daughter cells fuses with the hypodermis (hyp7) and the other daughter cells divides again at a later stage until it terminally differentiates at the L4 molt (Figure 6a) [16]. Seam cells of *lin-4(e912) *animals repeat the L1 fates in hypodermal cell lineages and are unable to exit the cell cycle at the L4 molt. In order to determine how seam cell development is altered in *lin-4 *knockdown animals, we followed the seam cell division pattern by observing GFP-labelled seam cells (wIs51, strain JR667). Both L1 and L2 divisions in *lin-4 *knockdown appeared to be normal, with each V cell (including V1 - V4 and V6) divided once in L1 (100%, *n *= 10, data not shown) and twice in L2 (100%, *n *= 10; Figure 6b, c). However, in early L3, V cells in *lin-4 *knockdown repeated the L2 division pattern, so that each V cell gave rise to twice as many daughter cells as the corresponding seam cells of the control animals (100%, *n *= 6; Figure 6d, e). This pattern of reiterated L2 seam cell division is consistent with a reduced, but not absent, activity of *lin-4*, possibly resulting in an intermediate level of *lin-14 *over expression [17].

Finally, since *lin-4 *functions through *lin-14*, mutations in *lin-14 *should suppress the phenotype of *lin-4 *knockdown. Indeed, at 20°C, inhibition of *lin-4 *in *lin-14(n179)*, a *lin-14 *nonnull mutant, with ^Rh^dextran-(^as-2'OMe^*lin-4*)_1 _(50 μM injection concentration) only caused Egl in 2.6% of labelled worms (*n *= 190) and all the examined young adult worms displayed normal alae (*n *= 30).

Together, these data showed that ^Rh^dextran-(^as-2'OMe^*lin-4*)_1 _caused developmental retardation consistent with *lin-4 *knockdown, confirming its efficacy and specificity in inhibiting *lin-4 *during development.

In order to test whether these conjugated antisense agents can be used to inhibit other miRNAs in worms, we prepared ^Rh^dextran-(^as-2'OMe^*lsy-6*)_1 _and ^Rh^dextran-(^as-2'OMe^*let-7*)_1 _using the same procedure as for making ^Rh^dextran-(^as-2'OMe^*lin-4*)_1_. These two dextran conjugates were designed to block *lsy-6 *and *let-7*, respectively, two miRNAs of known functions in *C. elegans*.

**Figure 7:**
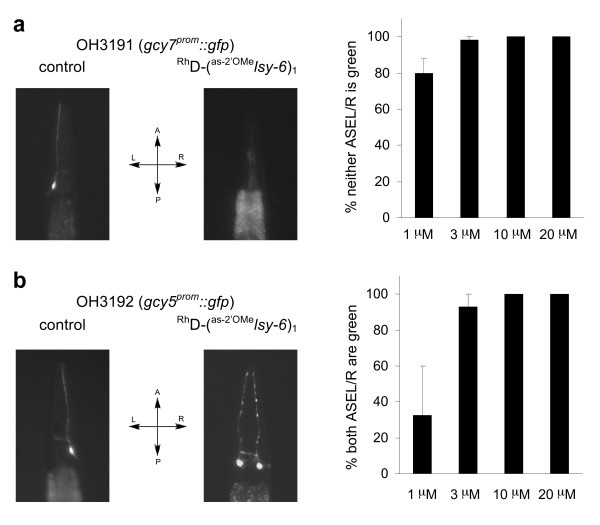
**Inhibit *lsy-6 *in neuronal cells with a dextran conjugated antisense reagent**. (a) Dose response of ^Rh^dextran-(^as-2'OMe^*lsy-6*)_1 _in repressing *gcy-7*^prom^*::gfp *expression in ASEL(left ASE). (b) Dose response of ^Rh^dextran-(^as-2'OMe^*lsy-6*)_1 _in causing ectotopic *gcy-5*^prom^*::gfp *expression in ASEL. L = left; R = right; A = anterior; P = posterior.

The *lsy-6 *microRNA regulates left-right asymmetry of ASE neurons, a pair of chemosensory neurons that share many bilaterally symmetrical features, yet differ in their ability to discriminate different ions by expressing distinct sets of chemoreceptors of the *gcy *gene family [18]. *lsy-6 *is only present in ASEL (left ASE) of adult worms. It promotes *gcy-7 *expression through repression of *cog-1*, a transcription factor that negatively regulates *gcy-7 *expression [18]. In order to score the inhibition of *lsy-6 *activity, we used two reporter strains expressing GFP in either ASEL (*gcy-7*^prom^*::gfp*, strain OH3191) or ASER (right ASE; *gcy-5*^prom^*::gfp*, strain OH3192). ^Rh^dextran-(^as-2'OMe^*lsy-6*)_1 _was highly effective in inhibiting *lsy-6*. At the injection concentration of only 3 μM, the compound inhibited *lsy-6 *with nearly 100% penetrance, assayed by the repression of *gcy-7*^prom^*::gfp *expression in ASEL (Figure 7a), and by the induction of ectopic *gcy-5*^prom^*::gfp *expression in ASEL (Figure 7b).

**Figure 8:**
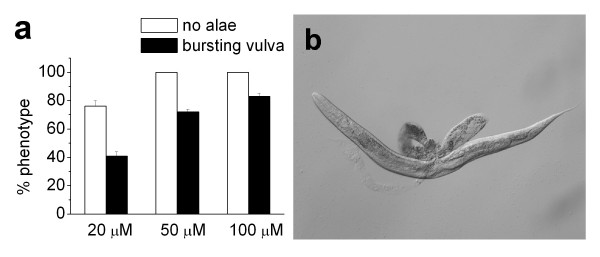
**Inhibit *let-7 *with ^Rh^dextran-(^as-2'OMe^*let-7*)_1_**. (a) Dose response of dextran-(^as-2'OMe^*let-7*)_1 _in causing bursting vulva or no alae formation. Error bars are standrad errors of two independent injections. After each injection, 50 or more embryos from injected worms were randomly picked and scored for bursting vulva or absense of alae when they reached adults. (b) An example image showing the bursting vulva phenotype.

Another miRNA, *let-7*, controls the larval-to-adult transition by repressing the translation of *lin-41 *and *hbl-1*. Mutations in *let-7 *lead to a retarded terminal differentiation of seam cells, which results in the elimination of alae and a bursting of the animal at the vulva as the animal undergoes the fourth molt [19]. We observed a dose-dependent bursting or no-alae phenotype, consistent with inhibition of let-*7 *activity in the progeny of hermaphrodites injected with ^Rh^dextran-(^as-2'OMe^*let-7*)_1 _(Figure 8). When ^Rh^dextran-(^as-2'OMe^*let-7*)_1 _was injected at 20 μM, it caused bursting vulva in about 40% of labelled worms and eliminated the formation of alae in nearly 80% of worms. Raising the concentration of ^Rh^dextran-(^as-2'OMe^*let-7*)_1 _to 50 μM or above increased the penetrance, and none of the labelled animals showed alae when they reached young adults. In contrast, an injection of ^Rh^dextran-(^as-2'OMe^*mir-84*)_1 _directed against another *let-7 *family microRNA caused no observable phenotype. Since *mir-84 *mutations do not cause visible phenotypes [20], this result is consistent with these antisense reagents inhibiting the targeted miRNA with high specificity. Further, in a *lin-41 *nonnull mutant, *lin-41(ma104)*, ^Rh^dextran-(^as-2'OMe^*let-7*)_1 _(50 μM) failed to produce the bursting vulva or alae defect in labelled worms (*n *= 12). This again suggested that these antisense reagents acted specifically by blocking their corresponding microRNAs.

**Figure 9:**
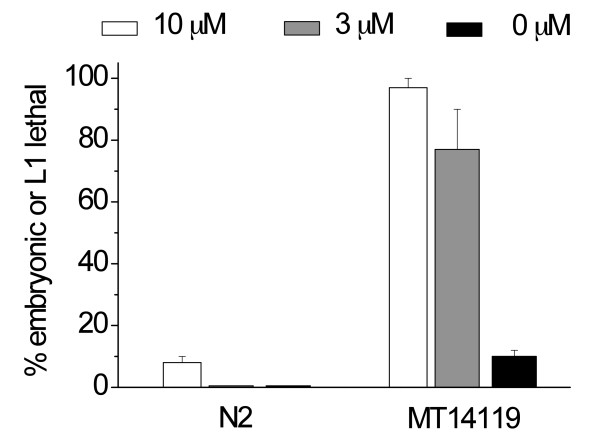
**Inhibit *mir-42 *with ^Rh^dextran-(^as-2'OMe^*mir-42*)_1 _during embryogenesis**. Error bars are standard errors of two independent experiments performed at 15°C. Each time we scored at least 50 worms labeled with an antisense reagent. MT14119 contains a deletion of 1261 bases on chromosome II which removes *mir-35 - mir-41 *(ref. [8]).

In order to confirm that this class of antisense reagents is also effective in inhibiting microRNAs during embryo development, we prepared ^Rh^dextran-(^as-2'OMe^*mir-42*)_1 _against *mir-42*, a member of the *mir-35 *family which consists of eight microRNA genes of similar sequences. This family of microRNAs is expressed only during embryogenesis [21] and functions redundantly to control embryonic development [8,22]. Deletion of the seven microRNAs (*mir35 - 41*, strain MT14119) leads to a temperature-sensitive late embryonic or L1 lethal phenotype. At 15°C, about 10% of the embryos displayed this phenotype, while the remaining 90% developed normally (Figure 9). In this *mir35-41 *null background, inhibition of *mir-42 *with ^Rh^dextran-(^as-2'OMe^*mir-42*)_1 _dramatically increased embryonic or L1 lethality: at the injection concentration of 3 μM, 77% of labelled worms were embryonic or L1 lethal. In contrast, inhibition of *mir-42 *had little effect in N2 strain (Figure 9).

**Figure 10:**
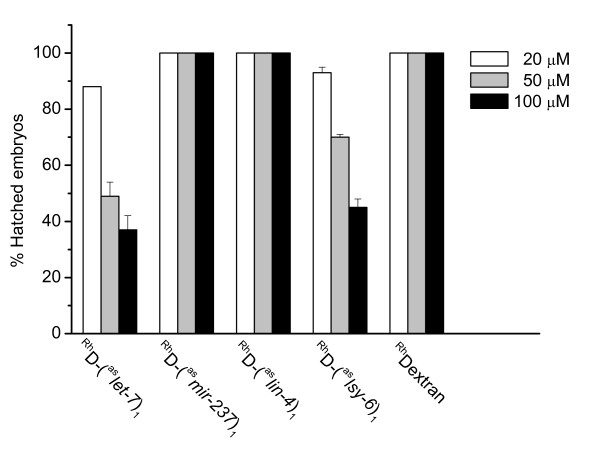
**Variation of percentage of hatched embryos labeled with different doses of antisense reagents**. Rhodamine-dextran (^Rh^Dextran) or its conjugates of antisense 2'-*O*-methyl oligoribonucleotides against *let-7*, *lin-4*, *lsy-6*, *mir-237 *were injected into gonads of N2 worms at three different concentrations. Each time we collected 50 or more labelled embryos and counted hatched larvae the next day. Error bars are standard errors of two independent injections. Examination of embryos that failed to hatch revealed that most of them were arrested around 50-cell stage. Embryos, once hatched, developed into adults that appeared to be grossly normal.

Among four miRNAs tested (*lin-4*, *lsy-6, let-7 *and *mir-42*), the dose of antisense reagents required for the effective inhibition of individual miRNA varied from as low as three micromolar (*lsy-6 *and *mir-42*) to as high as tens of micromolar (*let-7*). Two factors may account for the difference in the apparent potency of these antisense reagents. First, the cellular expression level of different miRNAs varies over a wide range [23], so a higher concentration of antisense reagents is needed to block more abundant miRNAs. Second, to inhibit a miRNA that is expressed late in larval development, it would require a higher concentration of reagent in the zygote to compensate for the dilution effect of cell division and larval growth. *let-7*, for example, is not expressed until the third larval stage and functions in the fourth stage [19]. By that time, the antisense reagent would be further diluted as animals grow and expand in size. In principle, a higher concentration of antisense reagents can be used to overcome the dilution effect. However, it should be cautioned that, as we raised the dose, we also noticed that an increasing number of embryos failed to hatch at concentrations above 50 μM. The extent to which these antisense reagents affect embryo development seemed to vary. Among the reagents tested, antisense reagents (antimirs) against *lin-4 *and *mir-237 *were best tolerated, with nearly 100% of the embryos hatched normally at 100 μM. By comparison, the antimir against *let-7 *was least tolerated, with close to 40% of embryos hatched at 100 μM (Figure 10). We have not yet explored the causes for this differential effect, although we anticipate that the future development of other bioconjugates employing different classes of oligonucleotides, for example locked nucleic acids or 2'-O-methoxyethyl oligoribonucleotides, may offer an improvement on the potency with concomitant reduction of the perturbation on embryo development.

**Figure 11:**
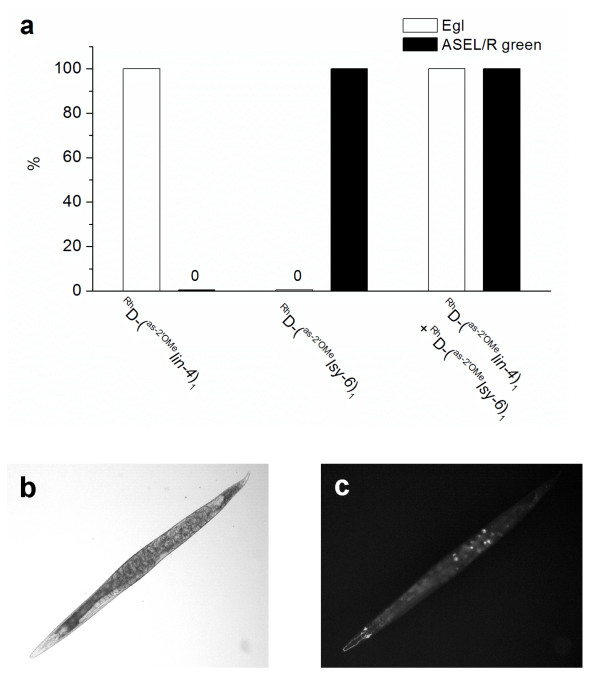
**Concurrent inhibition of *lin-4 *and *lsy-6 *in *Caenorhabditis elegans *with two antisense reagents**. (a) ^Rh^dextran-(^as-2'OMe^*lin-4*)_1 _(50 μM) or ^Rh^dextran-(^as-2'OMe^*lsy-6*)_1 _(20 μM), or a mixture of both reagents, were injected into gonads of OH3192 strain (*gcy-5*^prom^*::gfp*). The egg laying defective (Egl) and ectopic expression of *gcy-5*^prom^*::gfp *in ASEL (left ASE) were scored (*n *= 50 worms. Error bars are standard errors of two independent injection experiments). (b, c) Bright field [(b) showing Egl] and fluorescence [(c) showing ectopic expression of *gcy-5*^prom^*::gfp *in ASEL] images of a worm (OH3192) labelled with both ^Rh^dextran-(^as-2'OMe^*lin-4*)_1 _and ^Rh^dextran-(^as-2'OMe^*lsy-6*)_1_.

In order to test whether these antisense reagents can be used combinatorially to inhibit more than one miRNAs at a time, we coinjected ^Rh^dextran-(^as-2'OMe^*lsy-6*)_1 _and ^Rh^dextran-(^as-2'OMe^*lin-4*)_1 _into gonads of OH3192 strain (*gcy-5*^prom^*::gfp*). Together, these two reagents caused Egl in all labelled worms and they induced ectopic *gcy-5*^prom^*::gfp *expression in ASEL (Figure 11). In contrast, ^Rh^dextran-(^as-2'OMe^*lin-4*)_1 _alone did not alter the expression pattern of *gcy-5*^prom^*::gfp*, and ^Rh^dextran-(^as-2'OMe^*lsy-6*)_1 _by itself failed to cause Egl (Figure 11). The results again confirmed the specificity of these dextran-conjugated antisense reagents and suggested that multiple antisense reagents can be used in combination to block more than one miRNAs in *C. elegans*. As numerous deletion strains of single miRNA genes appear to be grossly normal, it has been suggested that redundancy might mask their functions [8]. Mixing these antisense reagents would allow the study of the combinatorial effects of multiple miRNAs on gene expression and should facilitate screening genetic interactions using mutants or reporter strains.

## Conclusions

We have developed a new class of antisense reagents that potently and selectively inhibit miRNAs in *C. elegans*. This offers an experimental approach complementary to mutational strategy for the study of the functions of miRNA *in vivo*.

## Methods

### General methods

2'-O-methyl oligoribonucleotides were either purchased from the Integrated DNA Technologies (IDT, Iowa, USA) or synthesized in-house by the standard solid phase phosphoramidite chemistry using an ABI 394 DNA/RNA Synthesizer (Applied Biosystems, California, USA). Sequences of 2'-O-methyl oligoribonucleotides used in this study are:

^s-2'OMe^*lin-4 *(sense): 5' - UCCCUGAGACCUCAAGUGUGA - 3'

^as-2'OMe^*lin-4 *(antisense): 5' - UCACACUUGAGGUCUCAGGGA - 3'

^as-2'OMe^*miR-237*: 5' - AGCUGUUCGAGAAUUCUCAGGGA - 3'

^as-2'OMe^*let-7*: 5' - AACUAUACAACCUACUACCUCA - 3'

^as-2'OMe^*lsy-6*: 5' - CGAAAUGCGUCUCAUACAAAA - 3'

^as-2'OMe^*miR-84*: 5' - UACAACAUUACAUACUACCUCA - 3'

^as-2'OMe^*miR-42*: 5' - UCUGUAGAUGUUAACCCGGUGA - 3'

For bioconjugation, an *n*-hexyl linker containing a disulfide bond (Thio-Modifier C6 S-S, Glen Research, Virginia, USA) was attached to the 5'-end of 2'-O-methyl oligoribonucleotides. A,C,G,U-2'-OMe-RNA CE phosphoramidite monomers and A,C,G,U-2'-OMe RNA synthesis supports were from AZCO Biotech (California, USA). MAL-dPEG_4 _™-NHS ester was from Quanta BioDesign Ltd (Ohio, USA). Dextran amine (40 KD) was purchased from Molecular Probes (Oregon, USA). Other reagents and solvents were from Aldrich (Missouri, USA). The UV and visible absorption spectra were recorded on a Shimadzu 2401 PC spectrometer.

### Conjugating 2'-O-methyl oligonucleotides with dextran

To prepare ^Rh^dextran-(^as-2'OMe^*lin-4*)_1_, for example, dextran amine (40 KD, ~ 8 amines/dextran, 10 mg) was first reacted with Rhodamine B isothiocyanate (RBITC, 0.4 mg, 0.75 μmol) in 0.1 mL anhydrous DMSO at 37°C for 8 h. MAL-dPEG_4_-NHS ester (3 mg, 5.84 μmol) was then added and the reaction was continued at room temperature overnight. The reaction mixture was dialyzed against water through a regenerated cellulose membrane (Float A Lyzer^®^, molecular wight cut off [MWCO] = 3500, Spectrum Laboratories, Inc. California, USA) to remove excess reagents. After freeze drying, the solid product was dissolved in water (0.25 mL) to make a 1 mM rhodamine-dextran stock solution.

To conjugate 2'-O-methyl oligoribonucleotides containing a 5'-disulfide (5' S-S) group with rhodamine-dextran, we first reduced the 5'-disulfide group to a free thiol using tris(2-carboxyethyl) phosphine (TCEP), a water soluble reducing reagent. ^as-2'OMe^*lin-4 *(5' S-S, 30 nmol) was dissolved in 100 μl of deaerated sodium phosphate buffer (100 mM, pH = 7.0). An excess amount of TCEP was added to the solution under the protection of Argon. One hour later, cold ethanol (0.5 mL) was added to precipitate the oligonucleotide. The supernatant was removed after centrifugation (14000 rpm for 10 min) and the precipitated oligonucleotide was redissolved in a sodium phosphate buffer (100 mM, pH 7.0, 70 μL). The oligonucleotide solution was then mixed with the rhodamine-dextran stock solution (30 μL) prepared above. The mixture was stirred under argon overnight. Excess mercaptoethanol was added to cap the remaining unreacted maleimide group. The reaction mixture was dialyzed against water using a cellulose membrane (MWCO = 10,000) and lyophilized to yield the final product. The dried product was re-dissolved in water to prepare a stock solution. The concentration of stock solution was typically in the range of 1 mM which was determined by measuring the UV absorption of 2'-O-methyl oligoribonucleotide at 260 nm. The UV absorption was converted to the oligonucleotide concentration using the OligoAnalyzer program accessible online http://www.idtdna.com/analyzer/Applications/OligoAnalyzer/. Rhodamine absorption at 260 nm was corrected according to its peak absorption at 559 nm. The conjugation yields were typically around 50%. The average stoichiometry of conjugation was calculated from the total amount of oligoribonucleotide in the final product divided by the amount of dextran added to the reaction.

^Rh^Dextran-(^as-2'OMe^*lin-4*)_4_, dextran-(^as-2'OMe^*lin-4*)_8 _and other dextran conjugates containing different sequences of 2'-O-methyl oligonucleotides were synthesized similarly. When preparing ^Rh^Dextran-(^as-2'OMe^*lin-4*)_4_, four equivalents of 2'-O-methyl oligonucleotides containing a 5'-disulfide group were used to react with the dextran-linked maleimide group. No Rhodamine B isothiocyanate was used when synthesizing dextran-(^as-2'OMe^*lin-4*)_8_.

Conjugation products purified by dialysis still contained a small amount of unconjugated oligonucleotides as analyzed by the polyacrylamide gel electrophoresis (PAGE), and were used for most of the experiments except for the ones shown in Figure 8 and Figure 10. To completely remove unconjugated oligonucleotides from dextran-conjugates, the reaction mixture was first concentrated under vacuum to a small volume (≤30 μL) and then mixed with 0.27 mL formamide (>99%, Ambion, Texas, USA). The mixture was boiled briefly and loaded into 5% preparative denaturing polyacrylamide gel. After running the gel at 400 V for 10 min, we confirmed the separation of free 2'-O-methyl oligoribonucleotides from dextran conjugates by viewing the gel over a fluorophore-coated thin-layer chromatography plate (Silica Gel 60, F254, Merck, Germany) under the UV illumination (265 nm). Dextran-conjugated products that remained near the origin of the gel showing red fluorescence were cut out and transferred into a dialysis membrane (MWCO = 1000) containing 2 mL of 0.5× TBE buffer (Bio-Rad, CA, USA). The dialysis membrane was sealed and the product in the gel was recovered by electrophoresis (300 V for 20 min). The TBE buffer in the dialysis membrane containing dextran conjugates was transferred to another cellulose dialysis membrane (MWCO = 10000) and dialyzed against water 3 times over 16 hrs to remove salts and urea. The final products were obtained as a powder after lyophilization.

### Worm injection and assay of miRNA inhibition in vivo

Dextran conjugates of 2'-O-methyl oligoribonucleotides were injected into both gonads of young adult worms of either wild-type (N2) or transgenics expressing GFP in an ASE neuron (OH3191 or OH 3192). Rhodamine dextran (40 KD, 8 mg/mL final concentration) was included in the injection solution if the injected reagents contained no fluorescent label, for example, dextran-(^as-2'OMe^*lin-4*)_8 _or unconjugated 2'-O-methyl oligoribonucleotides. For each experiment, we routinely injected a sample into ~ eight worms. About 16 h later, we collected rhodamine labelled embryos (*n *= 50) laid by injected worms under a fluorescence dissection scope (SteREO Discovery.V12, Carl Zeiss, Göttingen, Germany), and scored their phenotypes when they reached appropriate larval or adult stages. Staging of animal development was based on gonad size and morphology.

We also attempted delivering antisense reagents using a standard soaking method for RNAi ([24]. However, this method turned out to be ineffective. After incubating L1 larvae with ^Rh^dextran-(^as-2'OMe^*lin-4*)_1 _(300 μM) or ^Rh^dextran-(^as-2'OMe^*let-7*)_1 _(300 μM) for 48 h in the soaking solution (M9 solution (0.25 ×, without Mg^2+^) with 3 mM spermidine and 0.05% gelatin), we recovered L1 larvae on NGM plates. All the worms developed normally without showing any observable phenotype expected from *lin-4 *or *let-7 *knockdown.

## Abbreviations

ASEL: left ASE; ASER: right ASE; Egl: egg laying defective; GFP: green fluorescence protein; MWCO: molecular weight cut off; TCEP: tris(2-carboxyethyl) phosphine; UV: ultraviolet; 3'UTR: 3'-untranslated region.

## Competing interests

The authors declare that they have no competing interests.

## Authors' contributions

GHZ and WHL designed the conjugates and syntheses. VA and WHL designed biological assays. GHZ performed all the experiments. WHL conceived the project. WHL, VA and GHZ analyzed the data and wrote the manuscript.
